# Impact of transient acquired hypermutability on the inter- and intra-species competitiveness of *Pseudomonas aeruginosa*

**DOI:** 10.1038/s41396-023-01503-z

**Published:** 2023-09-04

**Authors:** Yue Yuan On, Wendy Figueroa, Catherine Fan, Pok-Man Ho, Éva Bernadett Bényei, Aaron Weimann, Christopher Ruis, Andres R. Floto, Martin Welch

**Affiliations:** 1 Department of Biochemistry, University of Cambridge, Cambridge, CB2 1QW UK; 2 Currently based at Epoch Biodesign, Oxford, UK; 3 Heart Lung Research Institute, University of Cambridge, Cambridge, UK; 4 University of Cambridge Molecular Immunity Unit, MRC Laboratory of Molecular Biology, Cambridge, UK; 5 Cambridge Centre for AI in Medicine, University of Cambridge, Cambridge, UK; 6 Department of Veterinary Medicine, University of Cambridge, Cambridge, UK; 7 Cambridge Centre for Lung Infection, Royal Papworth Hospital, Cambridge, UK; 8 Cambridge University Hospitals Trust, Cambridge, UK; 9 Department of Medicine, University of Cambridge, Cambridge, UK

**Keywords:** Bacterial genetics, Microbial ecology

## Abstract

Once acquired, hypermutation is unrelenting, and in the long-term, leads to impaired fitness due to its cumulative impact on the genome. This raises the question of why hypermutators arise so frequently in microbial ecosystems. In this work, we explore this problem by examining how the transient acquisition of hypermutability affects inter- and intra-species competitiveness, and the response to environmental insults such as antibiotic challenge. We do this by engineering *Pseudomonas aeruginosa* to allow the expression of an important mismatch repair gene, *mutS*, to be experimentally controlled over a wide dynamic range. We show that high levels of *mutS* expression induce genomic stasis (hypomutation), whereas lower levels of induction lead to progressively higher rates of mutation. Whole-genome sequence analyses confirmed that the mutational spectrum of the inducible hypermutator is similar to the distinctive profile associated with *mutS* mutants obtained from the airways of people with cystic fibrosis (CF). The acquisition of hypermutability conferred a distinct temporal fitness advantage over the wild-type *P. aeruginosa* progenitor strain, in both the presence and the absence of an antibiotic selection pressure. However, over a similar time-scale, acquisition of hypermutability had little impact on the population dynamics of *P. aeruginosa* when grown in the presence of a competing species (*Staphylococcus aureus*). These data indicate that in the short term, acquired hypermutability primarily confers a competitive intra-species fitness advantage.

## Introduction

Most lesions in DNA are repaired by the mismatch-repair machinery (MMR). When the MMR machinery is defective, for example due to mutation of the gene(s) encoding key components, this gives rise to an elevated rate of mutation—“hypermutation” [1]. In the opportunistic human pathogen, *Pseudomonas aeruginosa* (PA), mutations in genes encoding the MMR proteins, MutS and MutL, are the most frequent cause of hypermutability [2–5]. MutS is responsible for mismatch recognition, whereas MutL triggers excision of the mismatched base [6]. Hypermutators are particularly strongly associated with polymicrobial airway infections, such as those associated with cystic fibrosis (CF). In CF, around 60–90% of hypermutators contain loss-of-function mutations in *mutS* [2–5]. Loss of function mutations in *mutL* are also common, albeit slightly less so than *mutS* mutations. This presumably reflects the smaller size of *mutL* compared with *mutS* (ca. 1.9 vs 2.6 kb, respectively) rather than an intrinsic difference in the importance of each gene product to MMR; laboratory-generated deletion or insertion mutants in each gene exhibit comparably elevated mutation rates in vitro [4].

Hypermutability potentially allows cells to rapidly explore evolutionary solutions to ecological challenges (e.g., competition from co-habitants in a polymicrobial community) as well as environmental insults such as physical or chemical stress, and is therefore a powerful tool for studying how species adapt to ecological challenges. The problem is that once acquired, hypermutation is unrelenting and can lead to extensive collateral genomic damage and population heterogeneity. Therefore, if hypermutability is to be used as an experimental tool in adaptive laboratory evolution analyses (ALE) [7] or in experimental ecology (ExEc), we would ideally want to control it, and turn it “off” once a desired experimental outcome has been achieved (to prevent further detriment to the cell). In a previous study, Weigand and Sundin used a plasmid-borne, ultraviolet light-inducible error-prone Pol V polymerase to generate mutations in PA strain PAO1 [8]. Their elegant analysis revealed that the inducible UV-mediated hypermutator displayed an altered spectrum of single nucleotide polymorphisms (SNPs) compared with a PAO1 *mutS* mutant. However, in the Pol V system, the inducer (UV) is itself mutagenic, making this impractical as an experimental tool for the investigation of, for example, genetic adaptation in a polyspecies culture. The same argument would apply to the use of chemical mutagens to accelerate the mutation rate. More recently, over-expression of dominant-negative MMR-associated alleles (typically, *mutS* or *mutL*) from plasmids has been shown to be sufficient to hinder the native (endogenous) version of the protein from recognising DNA mismatches [9, 10]. However, while these approaches do indeed enable controlled hypermutability, they cannot reduce mutation rates to levels lower than that of the wild-type, and they require continual antibiotic exposure for plasmid maintenance.

**Fig. 1:**
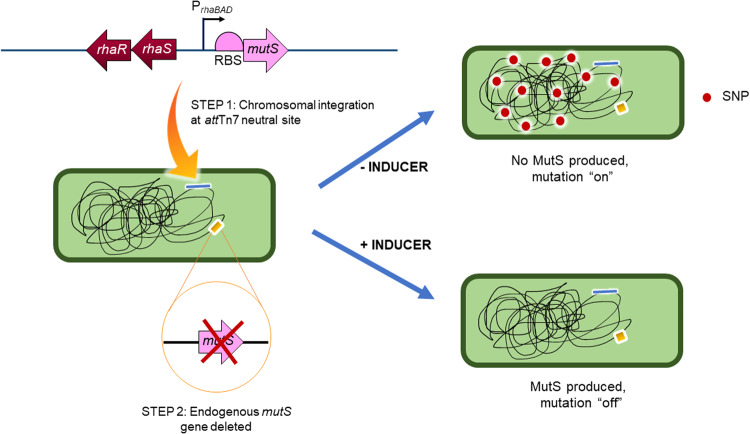
Principle behind the chromosomally-integrated inducible mutator strain. In step 1, a *mutS* transgene was introduced under the control of a rhamnose-inducible promoter at a neutral site in the PAO1 chromosome. In step 2, the endogenous *mutS* gene was deleted. This yielded a strain where, in the absence of inducer (rhamnose) mutability was high (due to low intracellular MutS levels) whereas in the presence of inducer, mutation is suppressed.

In the current study, we introduce a different approach to control the mutation rate in PA (Fig. 1). The rhamnose-inducible setup that we describe is chromosomally integrated and does not require selective antibiotics for maintenance. In addition to enabling us to increase the mutation rate to levels comparable to those seen in a *mutS* mutant, the setup also enables us to decrease the rate of mutation to levels much below that of the wild-type, essentially achieving a state of “hypomutation”. We exploit this fine level of temporal control to carry out a mutation-accumulation (“bottlenecking”) experiment, and whole-genome sequencing of the evolved isolates reveals an almost identical pattern of mutations to those seen in clinical *mutS* mutants. We also found that, in the short term, acquired hypermutability increases fitness relative to the wild-type progenitor. By contrast, acquired hypermutability had no impact on inter-species competitiveness.

## Material and methods

### Construction of the inducible mutator strain

PAYY01 was derived from PAO1. Briefly, pYY4S (containing the rhamnose-inducible *mutS* cassette, made as described in the *Supplementary Methods* section) was integrated at the *att*Tn7 neutral site in each recipient strain to generate PAO1-pYY4S-Gm^R^. Integration of the inducible system into the *att*Tn7 neutral site of the bacterial chromosome was confirmed by PCR using primers sets P_*glmS*-down_ and P_*Tn7R*_ (yielding a band size of 500 bp) or P_*glmS*-up_ and P_*Tn7L*_ (yielding a band size of 321 bp). The gentamicin resistance cassette was then excised using pFLP2-encoded flippase. Following this, pFLP2 was counter-selected using sucrose, yielding the gentamicin-sensitive strain PAO1-pYY4S-Gm^S^. Loss of the Gm^R^ cassette was confirmed by PCR amplification using primers Fwd (Gm^R^) and Rev (Gm^R^). We next deleted the endogenous *mutS* gene using a two-step allelic exchange [11], leaving the upstream and downstream open reading frames (PA3619 and *fdxA*, respectively) unaffected. The suicide plasmid (pEX18Gm_*mutS* K.O.) was introduced into the recipient strain by electroporation, and the cells were recovered in LB containing 30 mM rhamnose. Transconjugants were selected on LBA containing 50 μg/mL gentamicin supplemented with 30 mM rhamnose. Single colonies were then picked and struck onto low-salt LB-agar plates containing 15% sucrose and 30 mM rhamnose. The Gm^S^ sucrose^R^ phenotype of a selection of colonies was confirmed, and successful deletion mutants were verified by PCR using primers SP5.1 (PA3619) Fwd and *rpoS* IR Rev (Table S3). Successful deletion yielded a PCR product of 3138 bp, whereas wild-type revertants yielded a product of 5620 bp.

### Mutation accumulation (MA)

The MA procedure was a modification of previously described procedures [12, 13]. PAYY01 was revived from the frozen glycerol stock on LB-agar plates containing 30 mM rhamnose. A single colony was resuspended in 300 μL sterile PBS. Aliquots (10 μL volume) of this suspension were spotted onto 10 LB-agar (LBA) plates, or onto 10 artificial sputum medium agar (ASMA) plates, and struck to single colonies. In parallel, we also struck the cell suspension to single colonies on 3 plates of LBA and ASMA containing 6.25 mM rhamnose. Each of the resulting 26 plates was incubated for exactly 18 h at 37 °C. Following this, a single colony was picked from each plate and resuspended in 10 μL sterile PBS. From this cell suspension, 5 μL was struck onto a fresh plate of the corresponding medium and incubated again for 18 h at 37 °C. This process of bottlenecking was repeated for a total of 10 passages. After the 10th passage, a single colony of each lineage was cultured overnight in 5 mL LB supplemented with 30 mM rhamnose (to prevent further mutation) and genomic DNA (gDNA) was extracted for whole-genome sequencing. For comparative purposes, we also sequenced the genome of the ancestral strain, PAYY01.

### Polymicrobial competition assay

The polymicrobial continuous flow system was set up as described by O’Brien and Welch [14]. Briefly, overnight cultures of PAO1-Tc^R^ (PAO1 made Tet^R^ by integration of the miniCTX-based plasmid, pJM253 [15], at the *attB* site) and PAYY01 (grown in the presence of rhamnose), or of PAO1, PAYY01 and *Staphylococcus aureus* (SA; ATCC25923) were washed three times in sterile PBS. Each species was then used to inoculate 100 mL ASM ( ± rhamnose, as indicated) to an OD_600_ of 0.05. The ASM reservoir for the hypomutation experiment was supplemented with 50 mM rhamnose. The flow rate was 170 μL/min. Once the steady-state was established (typically, 24 h after inoculation), and when required, colistin was introduced into the reactor vessel by injection through the rubber-sealed port to a final concentration of 20 μg/mL. At designated time points, the pump was stopped temporarily, and 1 mL of sample was aseptically removed for CFU evaluation on Pseudomonas Isolation Agar (PIA) for PA, or Mannitol Salt Agar (MSA) for SA. When intra-species competition was being measured, PIA supplemented with 50 μg/mL tetracycline was used to discern PAO1-Tc^R^ from PAYY01.

## Results

### Construction of single-copy, genome-integrated inducible system

Our goal was to construct a chromosomally integrated system that allows cellular levels of MutS to be tightly regulated by an inert, exogenous inducer. After testing several inducible systems, we settled on pJM220, a miniTn7 delivery plasmid in which the expression of cloned genes comes under the control of the *rhaSR-*P_*rhaBAD*_ regulatory module [15]. Here, RhaR binds rhamnose and induces expression from the P_*rhaSR*_ promoter. This leads to the accumulation of RhaS in the cell. RhaS also binds rhamnose, and in doing so, activates transcription from the P_*rhaBAD*_ promoter. This regulatory setup appears to very effectively suppress – with negligible leakiness - the expression of genes cloned downstream of P_*rhaBAD*_ in the absence of rhamnose, yet also enables expression of the cloned gene to be modulated over a very wide range of induction ratios [15]. We confirmed that *P. aeruginosa* is unable to metabolise rhamnose (Fig. S1).

The PA *mutS* gene and its ribosome binding site (RBS) were inserted downstream of the P_*rhaBAD*_ promoter on pJM220 to yield pYY4S. We then integrated pYY4S into the PAO1 chromosome at the *att*Tn7 site. Following removal of the gentamicin-resistance cassette (Gm^R^) from the construct, we next used allelic exchange to delete the endogenous *mutS* gene, to yield strain PAYY01. In this construct, exogenous MutS levels (and hence, mutability) should be determined entirely by the concentration of rhamnose in the medium (Fig. S2). PAYY01 was generated and maintained (unless otherwise noted) in the presence of 30 mM rhamnose to maintain a state of genomic stasis.

### Mutability in PAYY01 is rhamnose-dependent

**Fig. 2:**
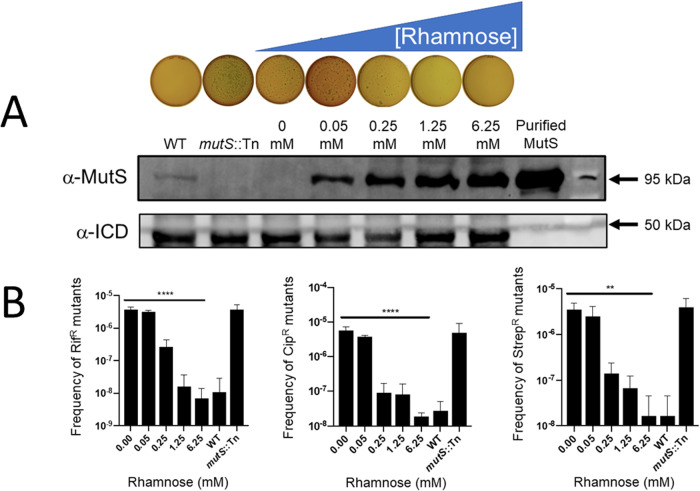
Rhamnose suppresses the mutation rate in PAYY01. **A** PAYY01 was grown overnight in LB containing the indicated concentration of rhamnose. The overnight cultures were then plated onto LBA containing the indicated concentration of rhamnose and 300 μg/mL rifampicin. Representative images of the plates are shown. In parallel, Western blotting was used to confirm that rhamnose induces MutS expression in the cultures. Antibodies raised against *iso*citrate dehydrogenase (ICD) were used as a loading control. **B** Quantitation of the mutation frequency in cultures challenged with rifampicin (300 μg/mL), ciprofloxacin (1 μg/mL), or streptomycin (500 μg/mL). **p ≤ 0.01; ****p ≤ 0.0001.

Resistance to certain antibiotics can arise due to mutations in key target genes. For example, rifampicin resistance is driven by the acquisition of point mutations in *rpoA*, *rpoB*, or *rpoC*. Therefore, and to test the ability of rhamnose to suppress mutability in PAYY01, we assayed the ability of rhamnose to suppress the appearance of mutations conferring resistance to rifampicin. The frequency of Rif^R^ isolates decreased as the [rhamnose] increased (Fig. 2A). In parallel, we monitored the expression level of MutS in the cultures using Western blotting. This revealed an inverse correlation between the level of MutS protein in the cell and the frequency of Rif^R^ mutants. We also note that the [MutS] required to elicit a wild-type frequency of Rif^R^ mutants was higher than the [MutS] in the wild-type cells. Possible reasons for this are addressed in the *Discussion*, below. In addition to rifampicin, point mutations can also give rise to resistance to ciprofloxacin (*via* mutations affecting *gyrA* or *parC*, or *via* mutations in *nfxB*, encoding a repressor of the *mexCD*-*oprJ* efflux pump) and streptomycin (mutations in *rpsL, rrs*, or *gidB*). For these antibiotics too, the mutation frequency decreased with increasing rhamnose concentration (Fig. 2B). Moreover, we note that in the absence of rhamnose, the mutant frequency on each of the tested antibiotics was comparable to that of a *mutS*::Tn mutant (PW7149) from the UWGC mutant library. By contrast, in the presence of 6.25 mM rhamnose, the mutation rate of PAYY01 was comparable to that of PAO1.

### High concentrations of rhamnose elicit *hypo*mutation in PAYY01

The highest rhamnose concentration we tested was 6.25 mM, yielding a PAYY01 mutation rate comparable to that of the wild-type (Fig. 2). We therefore wondered whether we might be able to depress the mutation rate further with higher concentrations of rhamnose, effectively achieving a state of hypomutation. This was indeed the case; when the concentration of rhamnose was increased to 50 mM, the Rif^R^ mutant frequency became ca. 4-fold (*p* ≤ 0.05) lower than that of the wild-type (Fig. S3).

### Whole-genome sequencing of evolved PAYY01 isolates indicate the same mutation spectrum as clinical *mutS* mutants

To understand better the impact of inducible mutability on genetic adaptation in PA at the single nucleotide level, we examined the mutational dynamics of PAYY01 in a mutation accumulation experiment (Fig. S4). PAYY01 was passaged through 10 distinct “evolutionary bottlenecks” on two different media (LB and artificial sputum medium) in either the absence or the presence of rhamnose. Passaging consisted of growth on an agar plate for 18 h to generate single colonies. A single colony from each plate was then streaked onto a new plate ( ± rhamnose, as appropriate) and the colonies were allowed to grow for another 18 h, and so on. After passage 10, a single colony from each lineage was subsequently grown in an aliquot of the respective medium containing 30 mM rhamnose to depress further mutation. Genomic DNA (gDNA) from each evolved isolate was extracted for whole-genome sequence analysis. For comparison, we also sequenced the genomic DNA of freshly revived PAYY01 that had always been maintained in the presence of 30 mM rhamnose. In parallel, we also measured the doubling time of PAYY01 on each medium (see *Methods*). This was done to enable a more accurate estimation of the mutation rate (per base pair per generation) from the sequence data.

**Fig. 3:**
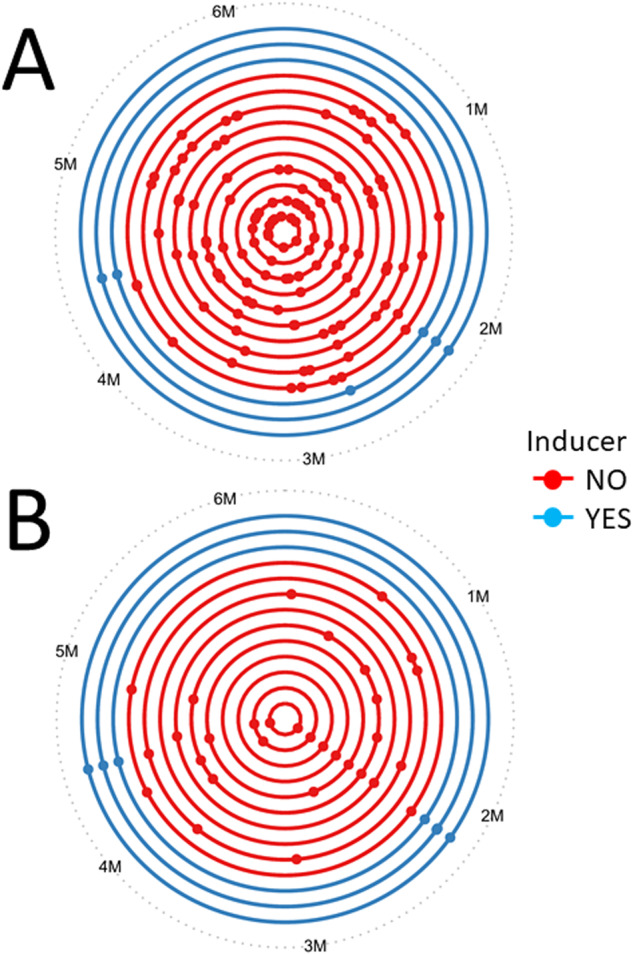
Mutation accumulation in PAYY01 grown on different media. Distribution of SNPs in the genome of evolved lineages of PAYY01 after 10 bottlenecks on (**A**) ASM-agar, and (**B**) LB-agar. Each circle represents the chromosome of a lineage of PAYY01. Dots represent the location of SNPs revealed by the whole-genome sequence analysis. Red and blue colours indicate lineages grown in the absence and presence of 6.25 mM rhamnose, respectively.

The bottlenecking experiment was done using two different media. The first, lysogeny broth (Lennox) agar (LBA), is a commonly used medium for propagating PA in the laboratory. The measured doubling time of PAYY01 during exponential growth on LBA was 46.9 min. The second was artificial sputum medium agar (ASMA), originally developed to mimic the nutritional environment in the CF airways. The measured doubling time of PAYY01 during exponential growth on ASMA was 53.2 min. The location of the SNPs/indels revealed by the whole-genome sequence data for the evolved lineages grown on each substrate are detailed in Table S4 and are summarised in Fig. 3. The most obvious feature of these data are that when mutability is “on” (i.e., in the absence of rhamnose), PAYY01 acquires far more SNPs/indels during growth on ASMA than it does on LBA. Based on the number of SNPs/indels acquired in the evolved lineages at the conclusion of passage 10, and on the measured doubling times in each medium, we were able to estimate the rate of mutation accumulation (Table 1). When *mutS* expression was induced in the presence of rhamnose, the rate of mutation accumulation in both LBA and ASMA medium was low and comparable (around 1.6 × 10^-9^ mutations/bp/generation in both media). However, the rate of mutation accumulation in the absence of rhamnose (i.e., mutability “on”) was >4-fold greater in ASMA than it was in LBA. This is not because ASM is inherently more “mutagenic” than LB; the frequency of Rif^R^ mutants, which should not be affected by growth in ASM, was the same in both media (Fig. S5).

**Table 1:** Mutation rate (mutations/bp/generation) of PAYY01 during growth on ASMA and LBA.

Medium	ASMA	LBA
No rhamnose present “hypermutation on” state	9.44 ± 1.53 × 10^−9^	2.15 ± 1.48 × 10^−9^
6.25 mM rhamnose present “hypermutation off” state	1.57 ± 0.79 × 10^−9^	1.62 ± 0.40 × 10^−9^

Data show the mean ± standard deviation.

**Fig. 4:**
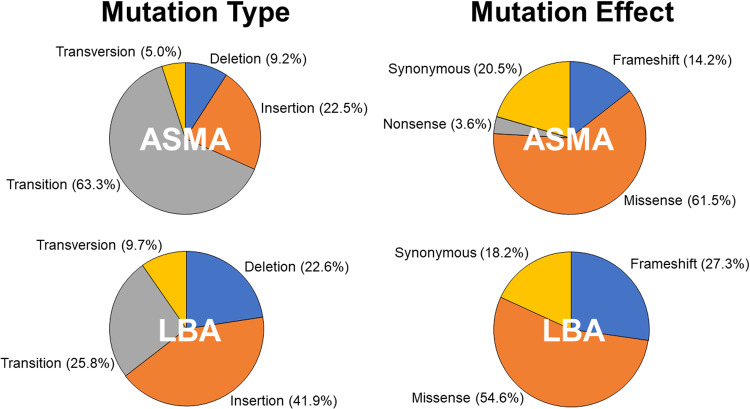
Pie charts displaying the mutation type and effect in samples grown on ASM agar and LB agar. Mutation type includes SNPs and indels in coding and non-coding parts of the genome. The total number of mutations identified in ASMA-grown isolates was 126. The total number of mutations identified in LBA-grown isolates was 38. The pie charts showing “mutation effect” include only SNPs and indels in coding regions.

Around 89% of the PAO1 genome is occupied by coding regions [16]. Following bottlenecking on ASMA, 69% of the 126 identified SNPs/indels were in coding regions. By contrast, just 39% of the 38 identified mutations in the LBA-grown cells were in coding regions. The majority of the mutations in ASMA-grown cells were transitions, followed by indels, whereas on LBA this trend was reversed and indels were more abundant than transitions. Transversion SNPs were relatively infrequent (<10%) in both cases. The type and effect of mutations in each medium is summarised in Fig. 4.

To further examine the mutational patterns in PAYY01 in the absence of rhamnose, we calculated the single base substitution (SBS) spectrum of the identified mutations (Fig. S6). The spectrum was dominated by C → T and T → C mutations, as expected from previous analyses [17], likely reflecting the error profile of DNA polymerase III (errors which are normally repaired by a functioning MMR system). Overall, the mutational pattern(s) were broadly similar to the previously calculated SBS spectrum of *P. aeruginosa mutS* mutants.

The SNPs/indels acquired following growth on ASMA were segregated into groups based on their Clusters of Orthologous Groups (COG) designation. After genes of unknown function, or genes for which only a general function could be assigned, the most highly populated COG categories were amino acid transport and metabolism (including ATP-binding cassette transporters), transcription, energy production and conversion, and inorganic ion transport/metabolism (Fig. S7). These functional gene categories are all commensurate with PAYY01 adapting to a new nutritional environment. For the LBA-grown samples, a smaller number of COG categories were represented (reflecting the smaller number of SNPs/indels overall in the samples) and no particular patterns of mutation acquisition were evident.

Most SNPs/indels appeared just once and independently in each lineage. However, SNPs/indels appeared at two loci (positions 2186927 and 4448855; PAO1 genome numbering) in several lineages. These loci are within non-coding regions and are associated with a GG → GCG insertion and a CG → GC transversion, respectively. They are likely spontaneous mutations that were acquired shortly after the experiment began (i.e., during passage 0) and were consequently inherited by subsequent generations. Note that these mutations were present in most but not all of the lineages, perhaps reflecting a heterogenous distribution within the colony selected for bottlenecking during this early passaging step. In a small number of cases, we also identified genes that had acquired different mutations in different lineages, suggesting that these genes may play an important role in adapting to the ASM environment. These mutations were in *nhaP* (PA3887, mutated independently in 2 lineages), *mexT* (PA2492, also mutated independently in 2 lineages), and *pdtA* (PA0690, mutated independently in 3 lineages).

### Growth in ASM and LB both yield a strong signal of negative selection

**Fig. 5:**
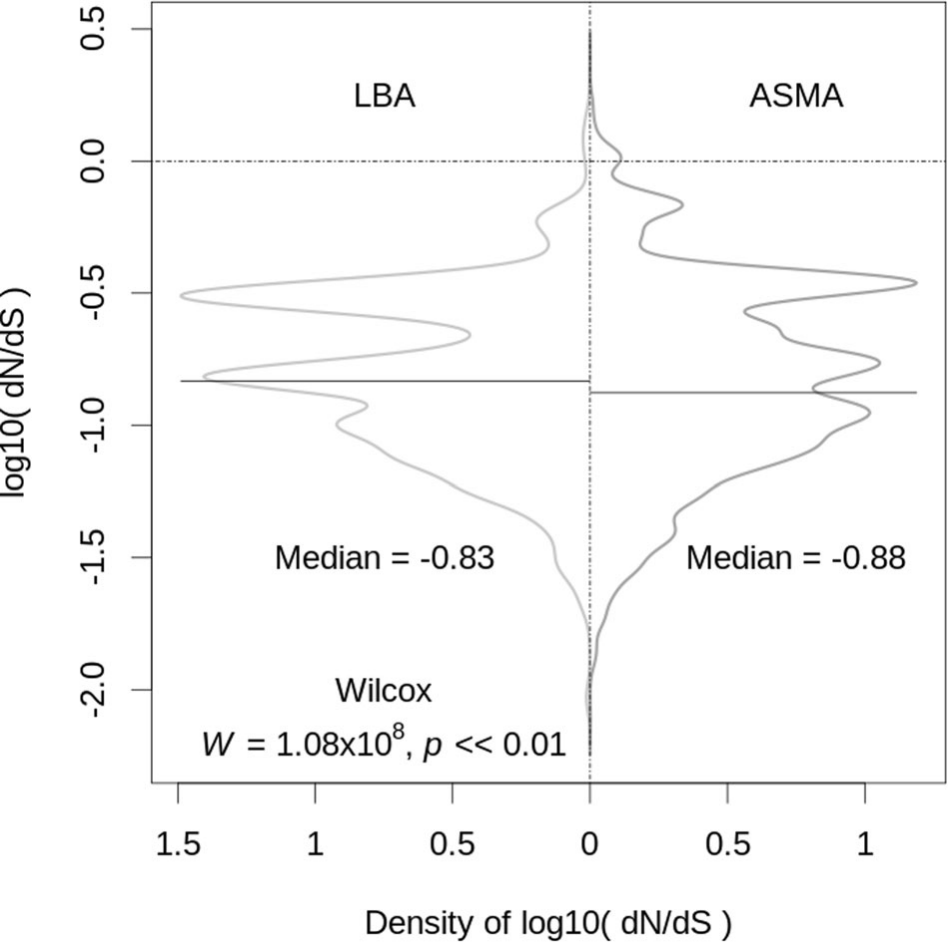
Distribution comparison of the log_10_(dN/dS) values between lineages selected on ASM and lineages selected on LB. The figure shows a density plot (with Gaussian assumption) of dN/dS for all 72 affected ORFs. Although the median values are similar, they are statistically different (*p* = 7.3 × 10^-7^) based on Wilcox’ test, as indicated. Multiple ORFs contributed to the peaks and troughs, although a small number of loci (PA3760, encoding a Pts system component (NagF); PA1740, encoding a hypothetical protein; and PA4100, encoding a likely dehydrogenase) appeared to be hotspots for selection.

The ratio dN/dS is a well-established indicator of likely selection pressure [18, 19]. A dN/dS value of 1 is indicative of drift, whereas a value of <1 is indicative of negative (“purifying”) selection. To calculate dN/dS for the SNPs identified following in vitro growth on ASM and LB, we first extracted the relevant affected ORFs from a selection of 854 isolates in the IPCD (International Pseudomonas Consortium Database), mostly obtained from people with CF. We then carried out a pairwise dN/dS calculation between the PAYY01-derived ASM- or LB-grown cells and the clinical isolates. This revealed that the ASM- and LB-grown lineages both displayed robust signatures of negative selection (median values of −0.88 and −0.83, respectively (Fig. 5)). However, the median dN/dS of the ASM-grown cells is significantly (*p* < 0.01) lower than that of the LB-grown cells, indicating that evolution in ASM (cf. LB) converges more towards that seen in clinical isolates.

### Acquisition of hypermutability confers a fitness advantage over wild-type *P. aeruginosa*

**Fig. 6:**
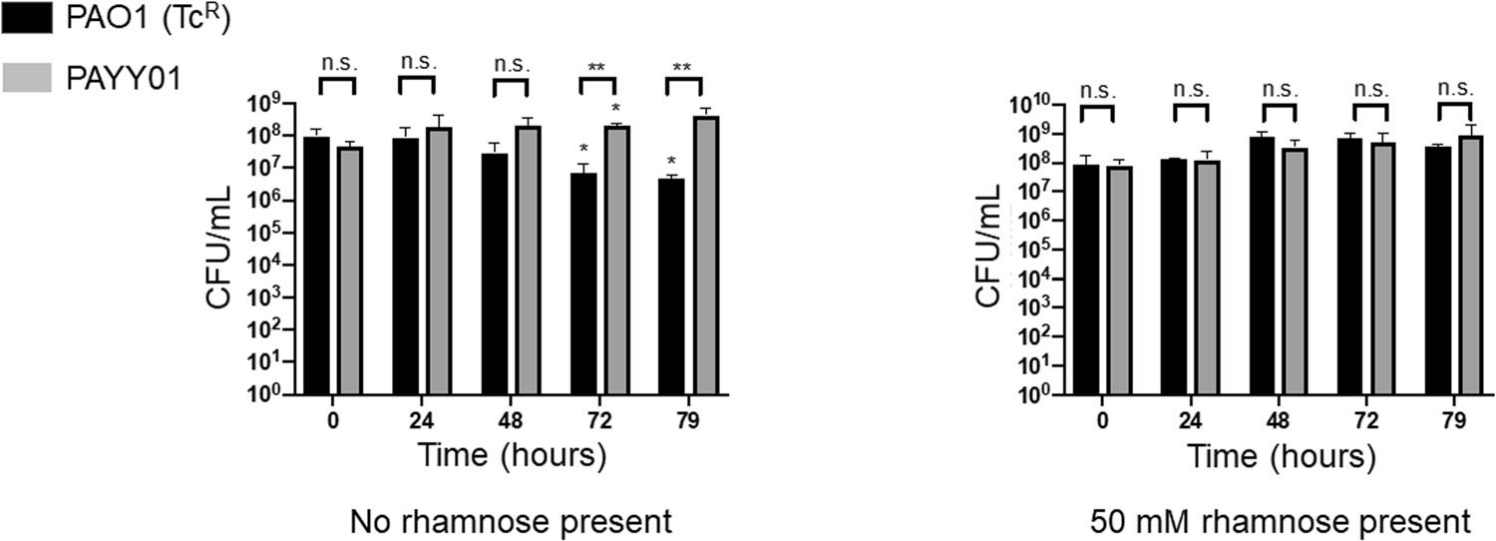
Wild-type *P. aeruginosa* is less fit than the inducible hypermutator lineage after four days of growth in ASM. Stable, steady-state mixtures of PAO1(Tc^R^) and PAYY01 were grown in the presence or absence of 50 mM rhamnose, as indicated. Samples were removed for strain enumeration at the indicated times. The data are the mean ± SD of three independent experiments. Asterisks within the body of the plots indicate significant differences relative to the 0 h data for the respective strain. Asterisks above each pairing in the plot indicate significant differences between members of each pair at that sampling point (*p ≤ 0.05, **p ≤ 0.01; n.s. *p* > 0.05).

In clinical scenarios, once acquired, hypermutators often rapidly sweep through a population [20, 21]. This is unexpected, because the unrelenting genomic “collateral damage” associated with loss of MutS function should lead to a fitness disadvantage. However, *mutS* mutants may potentially display enhanced fitness due to the acquisition of beneficial hitchhiking mutations [2]. To investigate the outcomes of this potential trade-off further, we co-cultivated a Tc^R^ variant of PAO1 with PAYY01 in an ASM-fed chemostat [14] in the presence of 50 mM rhamnose (to suppress mutation) or in the absence of rhamnose (to allow hypermutation). An engineered Tc^R^ variant of PA was used to enable facile discrimination between the wild-type and PAYY01 in plate assays. After 72 h of co-cultivation, we noted that PAYY01 displayed a distinct competitive advantage over PAO1(Tc^R^) in the absence of rhamnose (Fig. 6). This competitive advantage was manifested as a decline in the titre of PAO1(Tc^R^), whereas titres of PAYY01 were maintained at a relatively constant value. By contrast, in conditions that suppress mutability, PAYY01 and PAO1(Tc^R^) stably co-existed in equivalent titres. This indicates that the presence of the Tc^R^ cassette in PAO1(Tc^R^) does not incur a fitness cost per se.

**Fig. 7:**
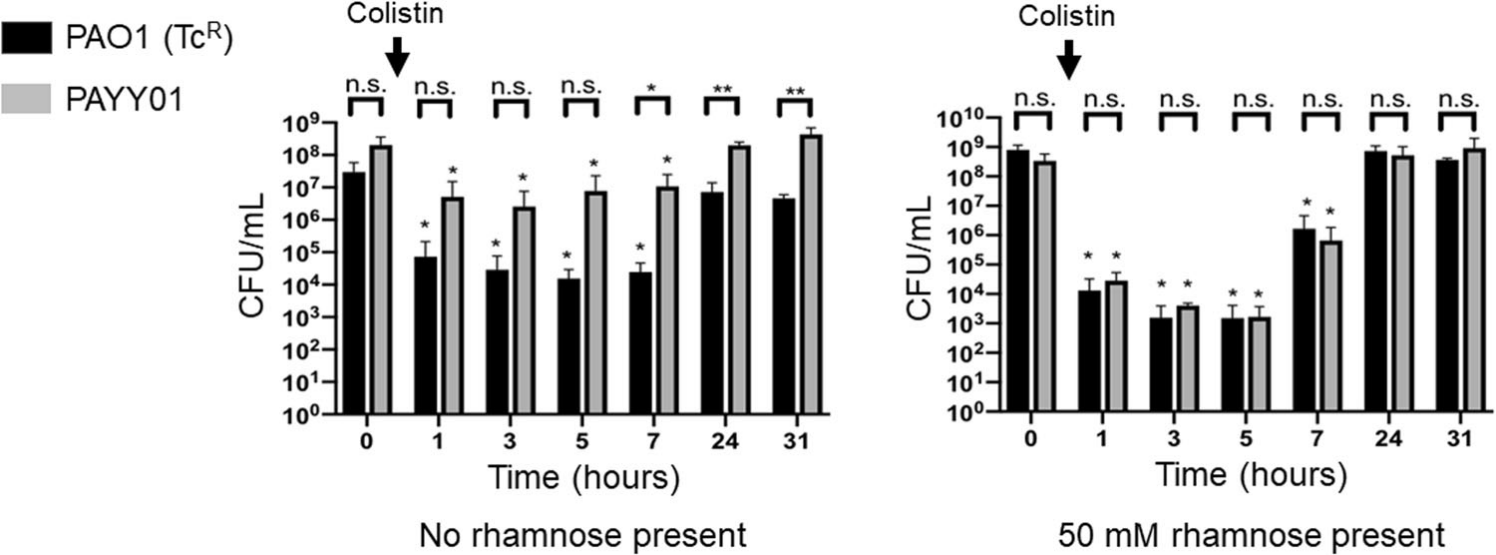
Population dynamics in mixed cultures of PAO1(Tc^R^) and PAYY01 upon challenge with colistin. Mixed cultures of PAO1(Tc^R^) and PAYY01 in ASM were allowed to reach a stable steady state for 24 h in the presence or absence of rhamnose, as indicated. The cultures were then challenged with a single dose of colistin (5 × MIC_PA_) at *t* = 0 h (corresponding to 24 h post-inoculation of the setup). Aliquots were withdrawn at the indicated times thereafter for CFU enumeration. *p < 0.05 (cf. the corresponding *t* = 0 h samples), n.s., not significant.

We next repeated the experiment above, except that following establishment of a steady state, the culture was challenged with a pulse of 5 × MIC_PA_ colistin (Fig. 7). This antibiotic is usually only prescribed when all other interventions fail, usually as a result of acquired multidrug resistance, so it was of interest to examine how hypermutation impacts on the response to this agent. In the presence of 50 mM rhamnose (to suppress mutation) titres of PAYY01 and PAO1(Tc^R^) both showed a comparable and precipitous decline (ca. 10^5^-fold) following colistin addition. Over the next few hours, titres recovered to pre-intoxication levels as the colistin was washed out of the system. By contrast, in the absence of rhamnose, PAYY01 titres showed a much smaller decline than those of PAO1(Tc^R^). Presumably, this reflects the presence of pre-existing pool of colistin-resistant mutants in the culture prior to challenge with this antibiotic. The relatively small impact of colistin addition on PAYY01 titres indicates that this pool of resistant mutants is likely quite large. Previous work has shown that colistin resistant mutants do readily arise in ASM, and are associated with mutations in the LPS biosynthetic genes [22].

### The acquisition of hypermutability by *P. aeruginosa* does not alter inter-species growth dynamics

**Fig. 8:**
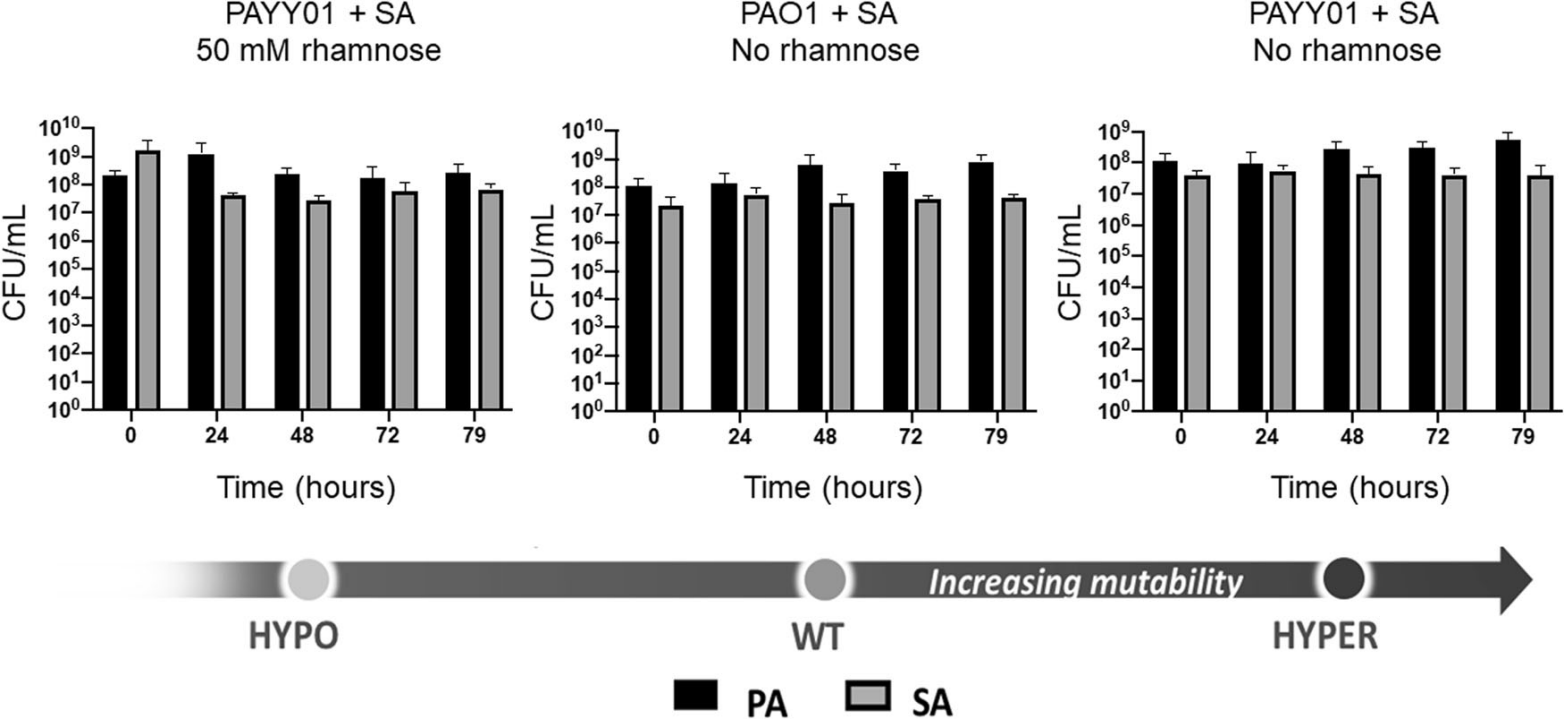
Acquired hypermutability does not affect inter-species dynamics in a PA-SA co-culture. A stable steady-state culture of SA and either PAO1 or PAYY01 was allowed to develop for 24 h in the presence or in the absence of 50 mM rhamnose (as indicated). Following this (“*t* = 0” in the figure), aliquots were withdrawn for enumeration of the two species on selective growth media at the indicated time points. The data are the mean ± SD of three independent experiments. In each panel, there were no significant (*p* ≤ 0.05) differences relative to the 0 h data.

Given that acquired hypermutability clearly influenced intra-species dynamics, we next wondered whether it might also influence inter-species dynamics. This is relevant since, in chronic infection scenarios such as the CF airways, PA hypermutators often share the niche with a variety of other species [23, 24]. Among the most frequently encountered co-habiting species in the CF airways is *Staphylococcus aureus* (SA) [25–27], and we have previously shown that PA and SA can be stably co-cultured in the continuous flow setup [14, 22, 28]. To investigate whether hypermutability affects PA-SA dynamics, stable steady-state co-cultures of PAYY01 and SA were set up in ASM ± 50 mM rhamnose, as previously described [14]. [We independently confirmed that SA does not metabolise rhamnose (Fig. S1)]. As a control, we also examined the inter-species dynamics in co-cultures of wild-type PAO1 and SA. Somewhat to our surprise, the acquisition of hypermutability by PA had no apparent impact PA-SA inter-species dynamics within the timescale of the experiment (Fig. 8).

## Discussion

In this manuscript, we investigate how the temporal acquisition of hypermutability affects inter- and intra-species dynamics in a medium formulated to mimic the chemical composition of the CF airways. In addition, PAYY01 also enables a state of induced *hypo*mutation to be achieved. Although this is not the first report of hypomutability per se - a state of hypomutability could be induced in *Escherichia coli* following over-expression of MutL [29] - to our knowledge, this is the first report of hypomutation being embedded as part of a graded response that also encompasses hypermutation.

The great advantage of the system described here is that the “starting state” culture is essentially homogenous and “wild-type”. This makes PAYY01 an excellent chassis for both experimental ecology (ExEc) and adaptive laboratory evolution (ALE) experiments [7]. Indeed, and provided that rhamnose is in constant supply, the genomic integrity of PAYY01 can be very stably maintained due to induced hypomutation. This is not the case with pre-formed *mutS* mutants, where genomic degradation is constant and unrelenting, leading to heterogeneity even within the initial inoculum.

One unexpected result was that when hypermutation was “on”, PAYY01 appeared to show a distinct fitness advantage compared with the wild-type, even in the absence of an overt selection pressure (Fig. 6). However, we note that (i) laboratory lineages of PAO1 have been maintained for decades on rich laboratory media such as LB, and have therefore likely acquired adaptations that favour growth in this medium, and (ii) the experiment in Fig. 6 was carried out in ASM, not LB. Exposure to this new medium is itself likely to impose a selection pressure. Acquired hypermutability would therefore be expected to increase the rate of adaptation to this new medium, accounting for the greater observed fitness cf. the wild-type. Presumably, the selection pressure associated with the transition from growth in LB to growth in ASM may also explain why the ASM-grown isolates fixed a greater number of SNPs in the bottlenecking experiments (Fig. 3). Perhaps more surprising was our observation that the population dynamics in a stable, mixed-species co-culture of *P. aeruginosa* and *S. aureus* were apparently unaffected (at least, within the time-frame of this experiment) by the acquisition of hypermutability in *P. aeruginosa*. This suggests that in the short-term – and starting from an essentially identical genomic template - acquired hypermutation initially favours intra-species adaptation over inter-species adaptation. This result was unexpected because it is now well-established that *P. aeruginosa* and *S. aureus* do interact and co-evolve in the CF airway environment [25–27]. Presumably, the compounded adaptive constraints imposed by exposure to both a new medium (ASM) and a co-habiting species (*S. aureus*) may limit the rate at which changes in inter-species dynamics manifest themselves. If so, in the longer-term - beyond the timescale that is feasible using our experimental setup - acquired hypermutability may well lead to altered inter-species dynamics. In this regard, Luján et al. have recently shown that *P. aeruginosa* mutability inversely correlates with microbial diversity in the CF airways [30]. This supports the notion that hypermutability might impact on inter-species interactions over longer periods. However, and as those authors note, there are also other, more prosaic explanations that may account for the correlation.

In the long-term, unrelenting hypermutation is likely to negatively impact *P. aeruginosa* fitness due to collateral genomic damage. Indeed, hypermutators isolated from people with CF have been demonstrated to display markedly reduced fitness (relative to paired, clonally-related normomutators) both in vitro and in vivo [3]. Moreover, this altered fitness has been shown to be due to the secondary mutations acquired as a result of the MMR defect [3]. This raises the question of why, in the current study, acquired hypermutability led to a fitness advantage over the wild-type. Presumably, in the face of a selection pressure, a short-term increase in the mutation rate is beneficial since it allows accelerated exploration of the “adaptational landscape” following challenge with e.g., a new growth environment (Fig. 6), or with an antibiotic (Fig. 7). Only in the longer-term, when additional, detrimental mutations get layered on top of these beneficial ones, will a fitness cost be incurred.

A key issue is whether the experimental setup described here is a good mimic for what happens in the CF airways. Although the in vitro setup is not a perfect mimic of what goes on in the CF airways (e.g., because it lacks immune input, has a lower polymicrobial diversity, and has a lower level of spatial heterogeneity than the CF airways) several factors suggest that it does capture key elements of CF-related hypermutator behaviour. First, ASM was used, mimicking the chemical environment of the airways. Second, the mutational signature in vitro was similar to that seen in CF-derived hypermutators. Third, the dN/dS signature was indicative of negative selection in action – as is the case with many CF-associated hypermutators. Finally, we note that several of the genes mutated in the ASM are also often mutated in CF isolates (e.g., *mexT*, genes encoding the type III secretion machinery, denitrification, and iron acquisition (Table S4)) indicative of a common selection pressure.

One of our more intriguing observations we made was that to achieve the same level of mutation suppression, MutS_PA_ needed to be expressed at much higher levels in PAYY01 than in PAO1 (Fig. 2). One possibility is that deletion of the endogenous *mutS* gene might have had a polar effect on a downstream ORF(s). In this regard, we note that the *mutS* ORF in PAO1 is operonic with the downstream *fdxA* ORF (Fig. S8). This raises the exciting possibility that *fdxA* might be required for optimal MutS activity in vivo. Recent discoveries from the Barton laboratory suggest that redox-active proteins involved in capturing electrons may well play a role in mismatch repair [31]. Another possibility is that MutL levels become limiting when MutS is over-expressed. Both possibilities are currently being explored.

We used PAYY01 to carry out a mutation accumulation (bottlenecking) experiment, comparing evolutionary trajectories following passaging on LB agar and ASM agar. The lineages grown on ASM acquired a four-fold larger number of SNPs/indels than those grown on LB. As noted above, a likely explanation for this is that growth in ASM is associated with a selection pressure, whereas growth in LB is associated with drift. Countering this argument, and somewhat unexpectedly, we noted that growth in ASM and LB both yielded a strong signature of negative selection in dN/dS analysis. However, only 8 SNPs from the LB-grown lineages qualified for dN/dS analysis, compared with 73 SNPs in the ASM-grown lineages, so the data here may be skewed by the small sample size of the LB-grown lineages. Consistent with the notion of a selection pressure being in operation in the ASM-grown cultures, most (69%) of the mutations in the ASM-grown cells were found in coding regions (where they would be expected to impact on gene function), whereas in the LB-grown cells the majority (61%) of mutations were located in intergenic regions, even though these regions constitute just 11% of the genome.

In summary, we present here a powerful tool for ExEc and ALE analyses, allowing fine control of mutability over a wide range of values. There is no reason why the regulatory circuit described here should not be adapted for use in other organisms. Indeed, we are currently attempting to engineer a similar regulatory circuit (under the control of a different inert chemical inducer) into *S. aureus*, allowing us to examine how the temporal induction of hypermutability in different species impacts on ecological interactions and adaptatory pathways.

### Supplementary information

Supplementary Material (data and methods)

Table S4

## Data Availability

All experimental data and R script coding are available in the GitHub repository (https://github.com/isaacyueyuan/PubPhD.git). The whole-genome sequencing data is available in the GEO repository (PRJNA955140).
