# Supplementary material for: Impact of transient acquired hypermutability on the inter- and intra-species competitiveness of Pseudomonas aeruginosa
Source: ISME J. 2023 Sep 4;17(11):1931–9. doi: 10.1038/s41396-023-01503-z (PMC10579334; doi:10.1038/s41396-023-01503-z)
Supplement: Supplementary file 1 — Supplementary Material (data and methods) [file 41396_2023_1503_MOESM1_ESM.docx]

**SUPPLEMENTARY FIGURES (pages 1-8)**

**SUPPLEMENTARY TABLES (pages 9-11)**

**SUPPLEMENTARY METHODS (pages 12-16)**

**SUPPLEMENTARY REFERENCES (page 17)**


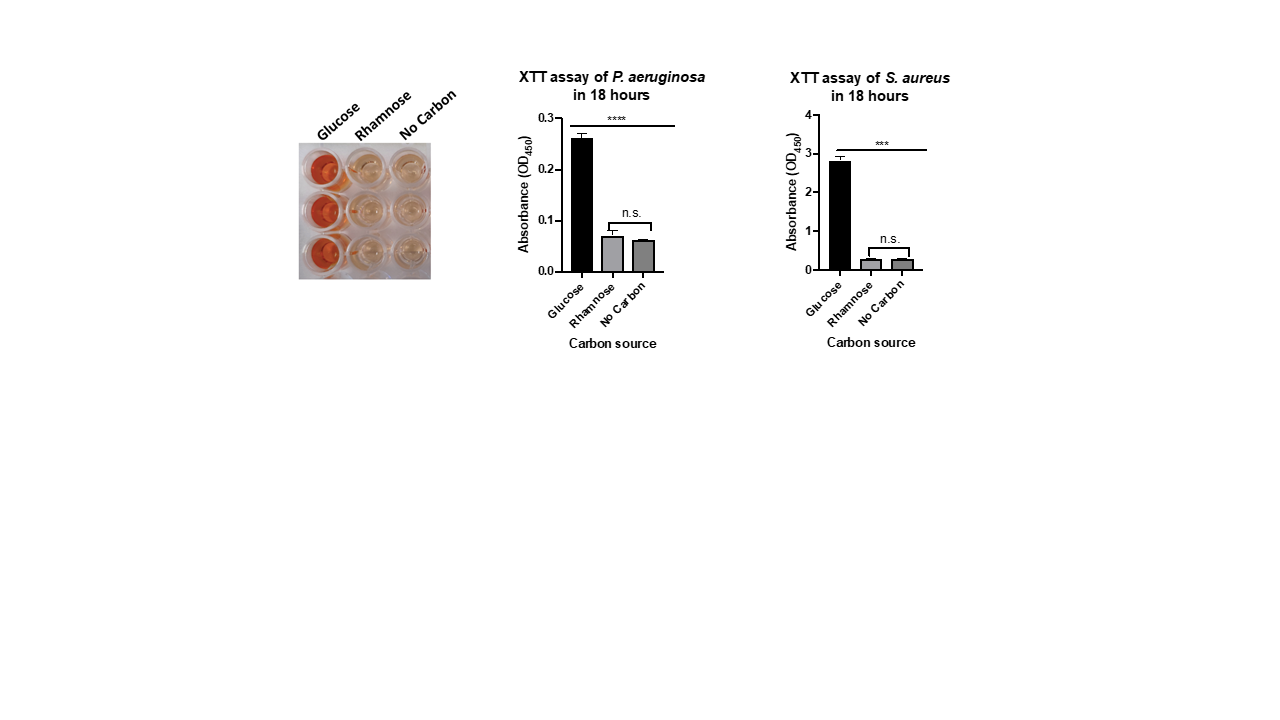


**Figure S1. Rhamnose is a non-metabolizable carbon source for *P. aeruginosa* and *S. aureus***. Aliquots of washed *P. aeruginosa* or *S. aureus* cell suspension were incubated with XTT and the redox cycling agent, menadione, in the presence of the indicated carbon sources. After 18 h, the A_450_ was measured.


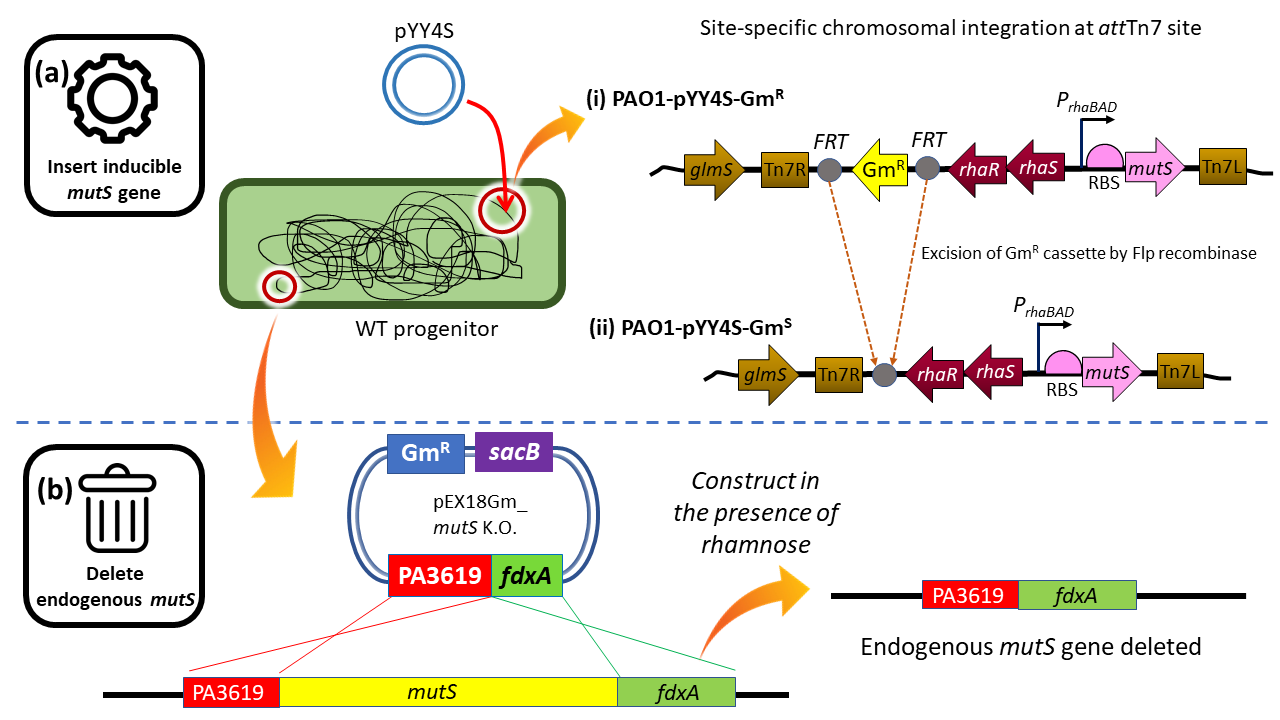


**Figure S2. Construction of PAYY01**. In step 1, pYY4S (from which the *mutS* ORF is expressed under the control of P_rhaBAD_) is inserted into a neutral site in the PAO1 chromosome. Next, the Gm^R^ cassette is deleted from the integrated pYY4S construct. Following this, and in the presence of rhamnose to ensure adequate mutation suppression, the endogenous *mutS* ORF is cleanly deleted using pEX18-mediated allele exchange.


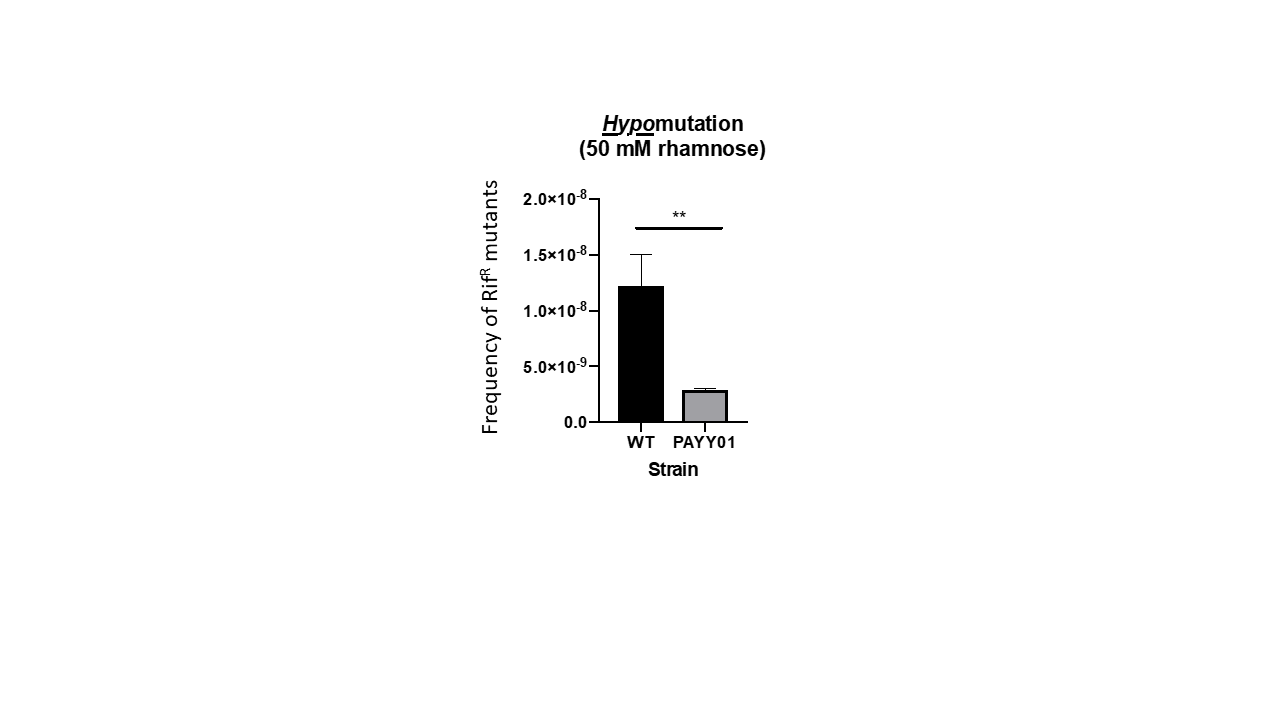


**Figure S3. Induced hypomutation in PAYY01 in the presence of 50 mM rhamnose.** The frequency of Rif^R^ mutants was calculated as the number of Rif^R^ colonies divided by the total number of CFU.


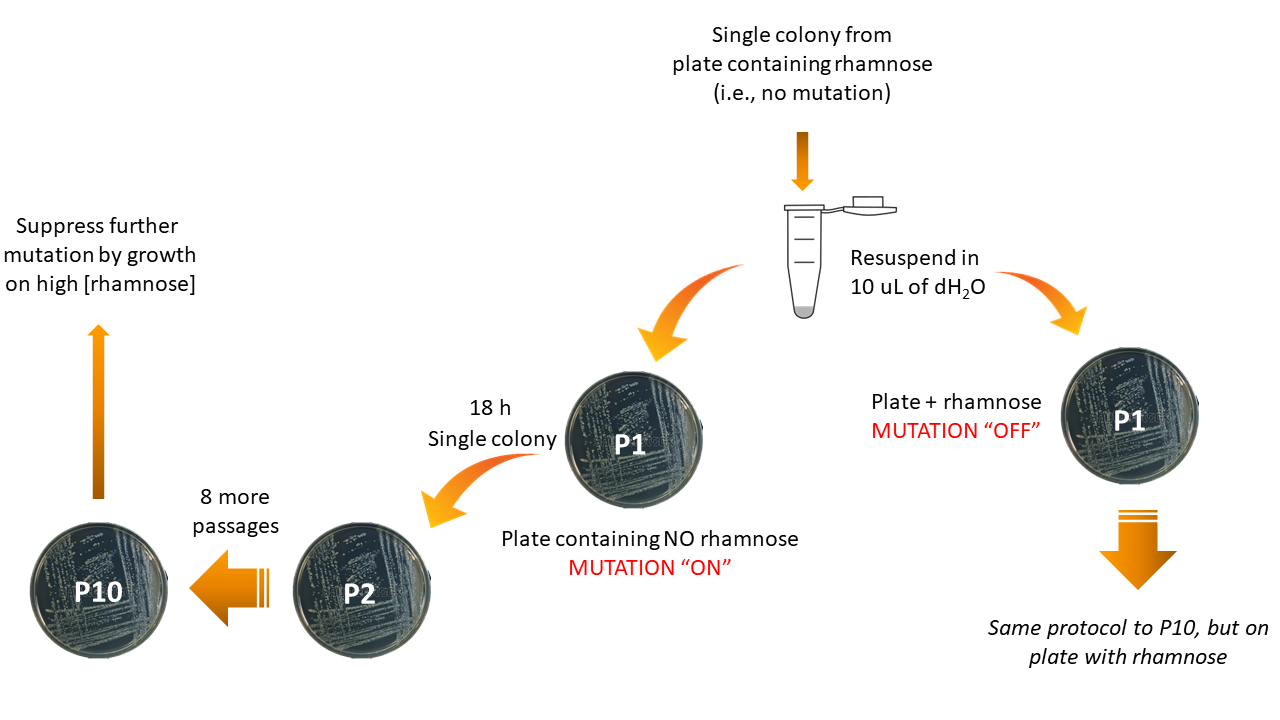


**Figure S4. Procedure for the mutation accumulation experiment**. PAYY01 was grown with ten successive “bottlenecking” steps on either ASM or LB agar ± 30 mM rhamnose. After 10 passages, an individual colony from each of 10 independent lines was grown up in the presence of 30 mM rhamnose and submitted for whole genome sequencing.


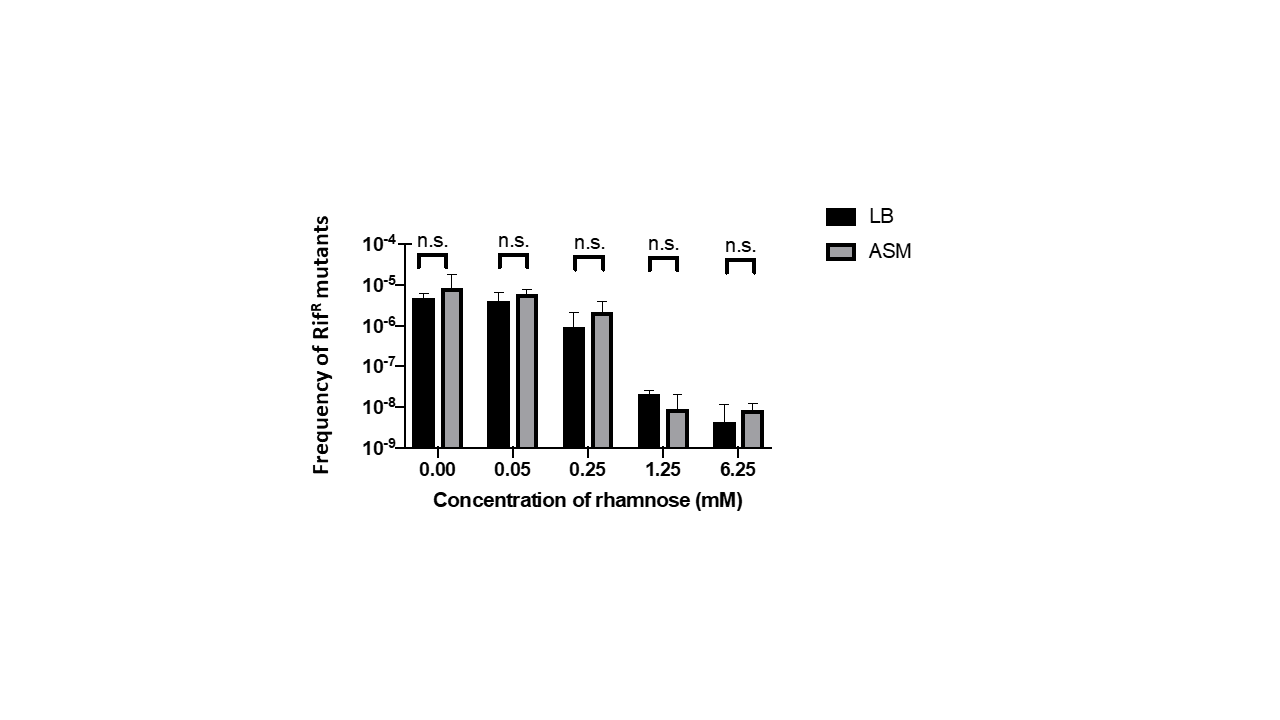


**Figure S5. The frequency of rifampicin resistant (Rif^R^) mutants is the same following growth of PAYY01 on ASM and LB**. ns = not significant (p > 0.05).


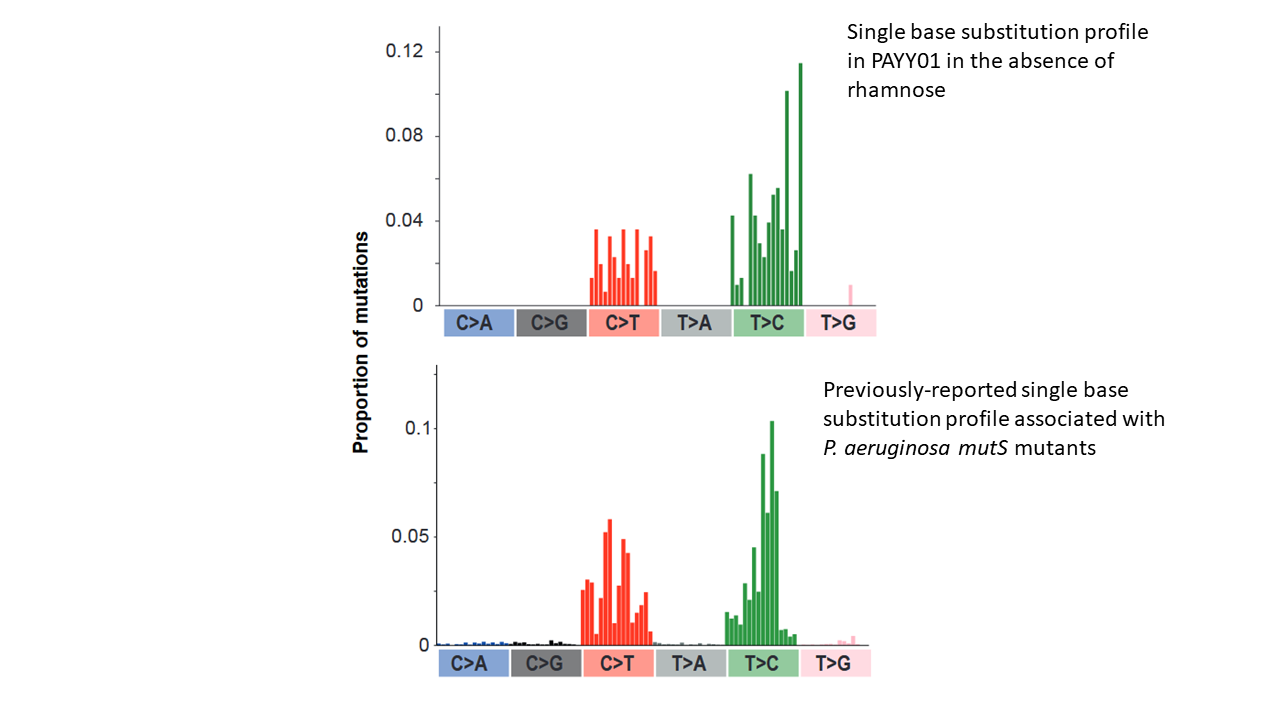


**Figure S6.** **Mutational spectrum in the absence of rhamnose.** The top panel shows the single base substitution spectrum (SBS) of mutations identified in samples bottlenecked in the absence of rhamnose. The six mutation types are shown in different colours, whereas the individual bars represent surrounding nucleotide contexts. The bottom panel shows the previously calculated SBS signature in *P. aeruginosa mutS* mutants for comparison.


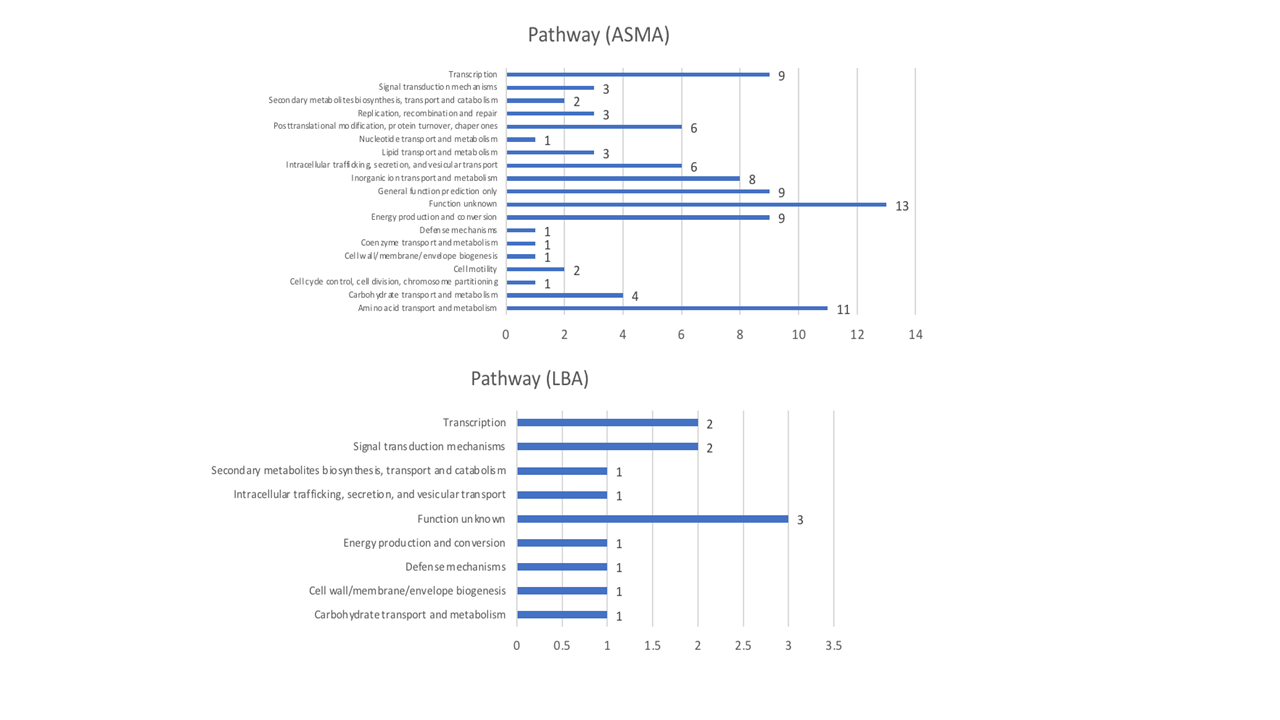


**Figure S7. COGs (clusters of orthologous genes) affected by SNPs and indels following growth on ASM agar (upper panel) and LB agar (lower panel)**.


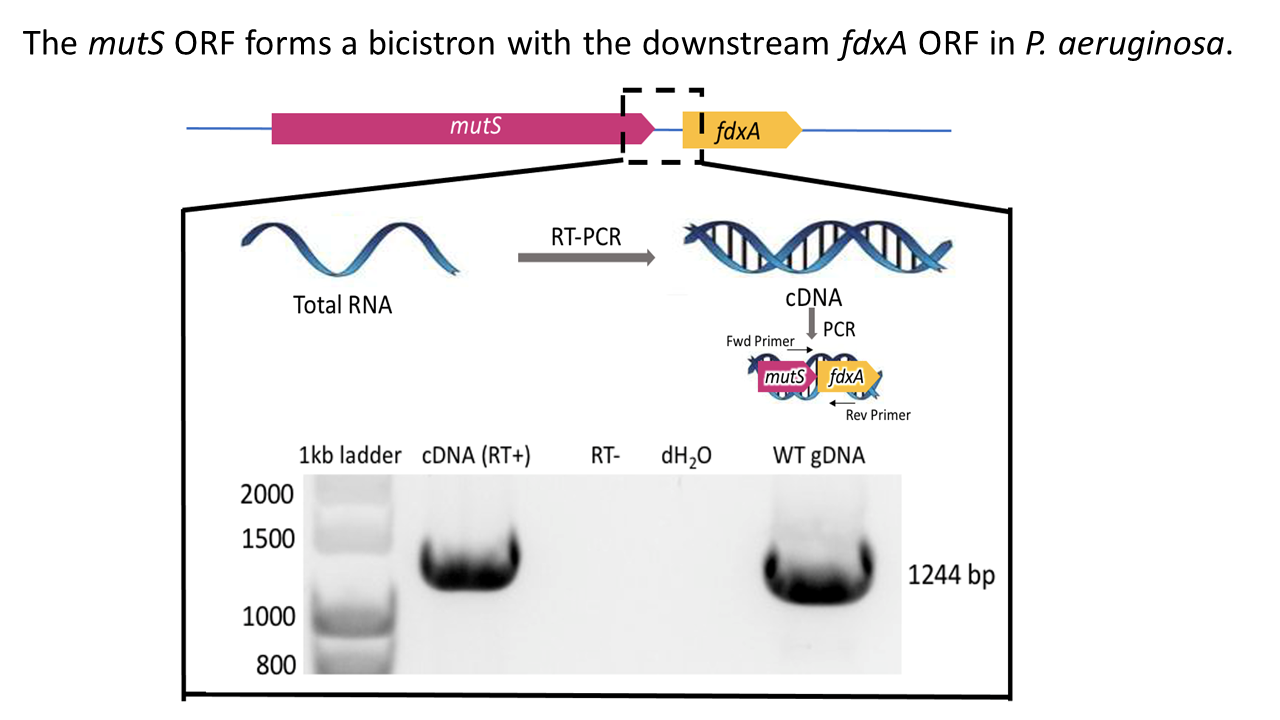


**Figure S8. The *mutS* ORF forms a bicistronic unit with the downstream *fdxA* ORF in *P. aeruginosa***. Total RNA was extracted from a mid-log phase culture of PAO1 and converted to cDNA using reverse transcriptase. The cDNA product was used as a template for amplification across the junction between the *mutS* and *fdxA* ORFs using primers 5’-TCGAACACACCTGCCTGCAC-3’ and 5’-GATTGGGCCCC GTATCAGCGCTCCAGATGC-3’. The size of the product obtained if the two ORFs are operonic would be 1244 bp. This is what was observed when cDNA (the RT+ sample) and gDNA were used as a template. No band was obtained in the negative control samples (RT- and dH_2_O). We conclude that *mutS* and *fdxA* are operonic.

**SUPPLEMENTARY TABLES**

**Table S1. Bacterial strains used in this study**

| **Strain** | **Description** | **Source** |
| --- | --- | --- |
| ***P. aeruginosa* (PA)** | | |
| PAO1 | Wild-type (WT) | [1] |
| PW7149 | UWGC MPAO1 *mutS*::IS*lacZ*/hah transposon mutant, Tc^R^, *mutS*::Tn | [1] |
| PAO1-pYY4S-Gm^R^ | PAO1 *att*Tn7::*rhaSR-P_rhaBAD_-mutS*, Gm^R^ | This study |
| PAO1-pYY4S-Gm^S^ | PAO1 *att*Tn7::*rhaSR-P_rhaBAD_-mutS*, Gm^S^ | This study |
| PAYY01 | PAO1 Δ*mutS* *att*Tn7::*rhaSR-P_rhaBAD_-mutS* | This study |
| PAO1-Tc^R^ | PAO1 *attB*::Tc^R^-*ori-int-oriT* | This study |
| ***E. coli*** | | |
| DH5α | (F^-^) *supE44 ΔlacU169* (φ*80lacZΔM15) ΔargF hsdR17 recA1 endA1 gyrA96 thi-1 relA1* | Gibco, BRL |
| β2163 | (F^-^) RP4-2-Tc::Mu_*dapA*::(*erm-pir*), Km^R^ Em^R^ | [2] |
| ***S. aureus* (SA)** | | |
| ATCC^®^ 25923™ | *S. aureus* subsp. *aureus* Rosenbach | American Type Culture Collection (ATCC^®^) |

**Table S2. Plasmids used in this study**

| **Plasmid** | **Description** | **Source** |
| --- | --- | --- |
| pJM220 | miniTn7 delivery vector with *rhaSR-P_rhaBAD_* inducible promoter (pUC18T-miniTn7T-Gm-*rhaSR*-*P_rhaBAD_*), Cb^R^ Gm^R^ | [3] |
| pJM253 | mini-CTX1 delivery vector with tetracycline resistance cassette (mini-CTX1-Tc*-rhaSR*-*P_rhaBAD_*), Tc^R^ | [3] |
| pYY4S | pJM220-derived vector with PA *mutS* gene insert and its RBS located downstream of *rhaSR-P_rhaBAD_* promoter (pUC18T-miniTn7T-Gm-*rhaSR*-*P_rhaBAD_*-*mutS*), Cb^R^ Gm^R^ | This study |
| pTNS2 | Helper plasmid which expresses Tn7 transposase for the integration of miniTn7-based plasmid into PA chromosome, Cb^R^ | [4] |
| pFLP2 | Site-specific FLP recombinase expression plasmid, Cb^R^ | [5] |
| pEX18Gm | Suicide vector for allelic exchange in knockout mutagenesis, Gm^R^ | [5] |
| pEX18Gm_*mutS* K.O. | pEX18Gm with flanking regions for *mutS* deletion, Gm^R^ | This study |

**Table S3. Primers used in this study**

| **Oligonucleotide** | **Sequence (5’ – 3’)** |
| --- | --- |
| HindIII Fwd (*mutS*) | ATATAAGCTTGCTTCCGAAGGCCCGTATGACC |
| PspOMI Rev (*mutS*) | GATTGGGCCCTCAGACCCGCATCTTCCATGCATATAAC |
| *rhaB* Fwd | CGTCGATTTTTCAAGATACAGCGTG |
| *mutS* Rev | GTTTTCGCTCAGCGCCTGCAGC |
| P*_glmS_*_-down_ | TTCCTATATCCATGCCGAAG |
| P*_Tn7R_* | CACAGCATAACTGGACTGATTTC |
| P*_glmS_*_-up_ | AACAACATCGCCATTCTCG |
| P*_Tn7L_* | ATTAGCTTACGACGCTACACCC |
| Fwd (Gm^R^) | TGTTAGGTGGCGGTACTTGGGTC |
| Rev (Gm^R^) | TGGAGCAGCAACGATGTTACGC |
| 5_*mutS* K/O Up-F (HindIII) | ﻿AATCAAGCTTTGCGCAGGAACCTGGAGAACGGC |
| 2_*mutS* K/O Up-R | GATCCTCGACAATTCATCGAACGGGCCTTCGGAAGC |
| 3_*mutS* K/O Down-F | GCCCGCTTCCGAAGGCCCGTTCGATGAATTGTCGAGGATCAATCCCG |
| 6_*mutS* K/O Down-R (BamHI) | GATCGGATCCGAAAAGGCCCGCTGATTGC |
| SP5.1 (PA3619) Fwd | ATGAACATCGCGCCACTCAAGG |
| *rpoS* IR Rev | GTTCGCCCGCCCACATCATG |
| *mutS* (Fwd) | TCGAACACACCTGCCTGCAC |
| PspOMI-*fdxA* (Rev2) | GATTGGGCCCCGTATCAGCGCTCCAGATGC |

**SUPPLEMENTARY METHODS**

**Strains and culture conditions.** The bacterial strains used in this study are listed in **Table S1**. All strains were routinely stored as glycerol stocks at -80°C and grown overnight on LB-Broth Lennox (Formedium) agar (LBA) at 37°C. The inducible mutator strain, PAYY01, was typically maintained in the presence of 30 mM rhamnose (unless otherwise stated) to maintain genome integrity. For plasmid maintenance in *Escherichia coli*, cultures were supplemented with 50 μg/mL carbenicillin, 15 μg/mL gentamicin or 10 μg/mL tetracycline, as indicated. For plasmid maintenance in PA, we used 250 μg/mL carbenicillin, 50 μg/mL gentamicin or 50 μg/mL tetracycline. A list of plasmids is shown in **Table S2**.

**Measurement of mutation rates.** Cultures of PAYY01 were prepared in LB containing 0.00 mM, 0.05 mM, 0.25 mM, 1.25 mM, 6.25 mM or 50 mM rhamnose, as indicated. Wild-type PAO1 and PW7149 (a *mutS* mutant from the UWGC PA two-allele library [1] containing a Tn insertion at position 1379/2168 in the ORF) served as controls. After 18 h of growth, the cultures were serially diluted and spread onto LB-agar ± antibiotic. The antibiotics were used at 300 μg/mL (rifampicin), 500 μg/mL (streptomycin) or 1 μg/mL (ciprofloxacin), as indicated [6–10]. When hypomutability was being assayed (i.e., in the presence of 50 mM rhamnose) the entire culture was pelleted and spread onto plates without serial dilution. The plates were incubated at 37°C for at least 18 h before recording the number of colony forming units.

**Whole-genome sequencing (WGS) analysis.** gDNA samples from the MA experiment were sequenced by MicrobesNG (Birmingham, UK) using Illumina technology (2 × 250 bp paired-end length reads, 30× depth). The WGS data analysis was performed essentially as described elswewhere [11]. Variant calling was performed using Snippy v2.5/Freebayes v0.9.21-7 with parameters: 10× read coverage, minimum base quality=20, and read concordance of 90% at a given locus. The variants were called relative to the relevant parental strain (PAYY01 or PAYY04). The reads were mapped with the reference genome (accession number [NC_002516](https://www.biorxiv.org/lookup/external-ref?link_type=GEN&access_num=NC_002516&atom=%2Fbiorxiv%2Fearly%2F2021%2F07%2F20%2F2021.07.20.453069.atom)) for visual inspection of called variants using Artemis [12].

**Calculation of *mutS* mutational signature.** Since the total number of mutations was small compared to the length of the genome, we assumed that it would be very unlikely that the same mutation would arise independently in multiple samples. We therefore filtered the data to only count each mutation once. The mutational spectra for the induced and non-induced samples were calculated independently with MutTui v2.0.2 (https://github.com/chrisruis/MutTui) using the filtered VCF files and the PAO1 reference. The resulting single base substitution (SBS) spectrum was rescaled based on the genomic composition of the PAO1 reference. To test whether the SBS spectrum of the non-induced samples was consistent with the previously reported *P. aeruginosa* *mutS* gene signature [13], we carried out 1000 down-samplings of the previous *mutS* gene signature and reduced this to 99 mutations (the number in the non-induced SBS spectrum).

**dN/dS analysis.** The 72 ORFs that uniquely acquired SNPs (as opposed to indels) during growth on LB or ASM in the mutation accumulation experiment were queried using BLAST (blastn v2.13.0) [14] against each of 854 annotated PA isolates (mostly from people with CF) in the International Pseudomonas Consortium Database (IPCD) [15]. From these BLAST searches, 87% of the ORFs matched (>99% identity) an ungapped alignment. We then calculated dN/dS (in all, 81130 calculations) for the hypermutator loci and for each equivalent clinical sequence. An unpaired Wilcox test was employed for sequence pairs containing both missense (dN > 0) and synonymous (dS > 0) mutations. BLAST and data sorting/formatting was carried out on the Cambridge Service for Data-Driven Discovery (CSD3) skylake-himem partition with bash v4.2.46(2)-release. The dN/dS calculations and statistical tests were carried out with R v4.1.2.

**Plasmid construction.** To construct pYY4S, we PCR-amplified *mutS* (including its ribosome binding site) from PAO1 using the primers HindIII Fwd (*mutS*) and PspOMI Rev (*mutS*). A list of the primers used in this work is shown in **Table S3**. Following digestion with HindIII and PspOMI, the amplicon was introduced into appropriately-digested pJM220. The insert was confirmed by sequencing using primers *rhaB* Fwd and *mutS* Rev.

To construct the suicide plasmid, pEX18Gm_*mutS* K.O., we used a two-step PCR. In the first reaction, ca. 500 bp region upstream of the *mutS* gene in PAO1 were amplified using primers 5_*mutS* K/O Up-F (HindIII) and 2_*mutS* K/O Up-R. In parallel, the ca. 500 bp region downstream of *mutS* was amplified using primers 3_*mutS* K/O Down-F and 6_*mutS* K/O Down-R (BamHI)*.* The upstream and downstream fragments were then used as templates for splicing-by-overlap extension (SOE) PCR with primers 5_*mutS* K/O Up-F (HindIII) and 6_*mutS* K/O Down-R (BamHI) [16]. The product was digested with HindIII and BamHI and introduced into pEX18Gm.

Plasmids were introduced into recipient strains by electroporation, as previously described [17]. For the plasmids destined for chromosomal-integration, we co-electroporated a helper plasmid, pTNS2, alongside the mini-Tn7 plasmid (pYY4S).

**Measurement of rhamnose metabolism**. The metabolic activity of PA and SA was quantified by monitoring XTT (sodium 3´-[1-(phenylaminocarbonyl)-3,4-tetrazolium]-*bis*(4-methoxy 6-nitro) benzene sulfonic acid hydrate) reduction in the presence of rhamnose or glucose (or, as a control, no added carbon source). Briefly, overnight cultures of PA and SA were washed 3 × with sterile PBS and resuspended in 10 mL of PBS. The cell suspensions were then diluted in an M9 liquid medium with 28 mM glucose or 28 mM rhamnose (or, no carbon source) to an initial OD_600_ of 0.05. This suspension was dispensed in 100 μL aliquots into a 96-well plate (5 replicates for each carbon source). After 2.5 h at 37°C, 100 μL of XTT-menadione solution (0.4 mg/mL XTT and 10 μM menadione) was added to each well. The plate was sealed with a sterile breathable membrane (StarLab) and incubated for a further 18 h at 37°C in the dark. After this, the absorbance in each well was measured at 450 nm (to measure released formazan) and 660 nm (to monitor cell density) in a FLUOstar^®^ Omega microplate reader (BMG Labtech). Bioactivity was measured as: [A_450_ (sample) – A_450_ (blank)] – A_660_ (sample).

**Western blotting.** Protein samples were resolved by SDS-polyacrylamide gel electrophoresis and then transferred onto 0.45 µM immobilon-P PVDF membranes (Millipore). For detection of MutS, the membranes were incubated with a polyclonal anti-MutS primary antibody preparation (1:2500 dilution). [The antibodies were raised in rabbits against purified His_6_-tagged MutS, and were pre-adsorbed against acetone-precipitated *mutS* mutant (PW7149) prior to use.]. Antibodies raised against a housekeeping protein, *iso*citrate dehydrogenase (ICD), served as a control (1:15000 dilution). The blots were developed using IRDye^®^ 800CW goat anti-rabbit IgG secondary antibody (LI-COR Biosciences) (1:15000 dilution). Signals were detected using a ChemiDoc Imaging System (Bio-Rad).

**Doubling time measurement for surface growth.** An overnight culture of PAYY01 was washed 3× with sterile PBS and then diluted to OD_600_ 0.05. This was designated as the 0^th^ h sample. Ten plates each of LBA and ASMA were prepared and a sterile 0.22 μm nitrocellulose membrane filter (Millipore) was laid onto the agar surface of each. An aliquot (100 μL volume) of cell suspension was spotted onto each membrane, and the timer was started. The plates were incubated at 37°C. Over the next 20 h, and at 2 hourly intervals, an inoculated membrane was removed and placed into 10 mL sterile PBS. Cells were dislodged by vortexing the sample for 1 min. The cell suspension was serially diluted and spread onto LBA plates in technical replicates. CFU counts were evaluated the following day. For growth on the two media, three biological replicates were carried out.

**Statistical analyses.**

Unless otherwise stated, statistical analyses were done using GraphPad Prism version 8.1.0. Typically, analyses involved an unpaired *t*-test based on two-tailed *p* value, or (if multiple comparisons were being made) ANOVA. The data for the polymicrobial cultures were analyzed using pairwise *t*-tests with Bonferroni *p* value adjustment. The False Discovery Rate (FDR) was controlled using the Benjamini/Hochberg step-up procedure (BH) [18] and Storey’s *q* value approach [19].

**SUPPLEMENTARY REFERENCES**

1. Jacobs MA, Alwood A, Thaipisuttikul I, Spencer D, Haugen E, Ernst S, et al. Comprehensive transposon mutant library of *Pseudomonas aeruginosa*. *Proc Natl Acad Sci* 2003; **100**: 14339–14344.

2. Demarre G, Guérout AM, Matsumoto-Mashimo C, Rowe-Magnus DA, Marlière P, Mazel D. A new family of mobilizable suicide plasmids based on broad host range R388 plasmid (IncW) and RP4 plasmid (IncPα) conjugative machineries and their cognate *Escherichia coli* host strains. *Res Microbiol* 2005; **156**: 245–255.

3. Meisner J, Goldberg JB. The *Escherichia coli rhaSR-PrhaBAD* inducible promoter system allows tightly controlled gene expression over a wide range in *Pseudomonas aeruginosa*. *Appl Environ Microbiol* 2016; **82**: 6715–6727.

4. Choi K-HH, Gaynor JB, White KG, Lopez C, Bosio CM, Karkhoff-Schweizer RAR, et al. A Tn7-based broad-range bacterial cloning and expression system. *Nat Methods* 2005; **2**: 443–448.

5. Hoang TT, Karkhoff-Schweizer RR, Kutchma AJ, Schweizer HP. A broad-host-range F1p-FRT recombination system for site-specific excision of chromosomally-located DNA sequences: Application for isolation of unmarked *Pseudomonas aeruginosa* mutants. *Gene* 1998; **212**: 77–86.

6. Oliver A, Cantón R, Campo P, Baquero F, Blázquez J. High frequency of hypermutable *Pseudomonas aeruginosa* in cystic fibrosis lung infection. *Science (80- )* 2000; **288**: 1251–1253.

7. Oliver A, Baquero F, Blazquez J. The mismatch repair system (*mutS, mutL* and *uvrD* genes) in *Pseudomonas aeruginosa*: Molecular characterization of naturally occurring mutants. *Mol Microbiol* 2002; **43**: 1641–1650.

8. Mandsberg LF, Ciofu O, Kirkby N, Christiansen LE, Poulsen HE, Høiby N. Antibiotic resistance in *Pseudomonas aeruginosa* strains with increased mutation frequency due to inactivation of the DNA oxidative repair system. *Antimicrob Agents Chemother* 2009; **53**: 2483–2491.

9. Morero NR, Monti MR, Argaraña CE. Effect of ciprofloxacin concentration on the frequency and nature of resistant mutants selected from *Pseudomonas aeruginosa mutS* and *mutT* hypermutators. *Antimicrob Agents Chemother* 2011; **55**: 3668.

10. Morero NR, Argaraña CE. *Pseudomonas aeruginosa* deficient in 8-oxodeoxyguanine repair system shows a high frequency of resistance to ciprofloxacin. *FEMS Microbiol Lett* 2009; **290**: 217–226.

11. O’Brien TJ, Figueroa W, Welch M. Decreased efficacy of antimicrobial agents in a polymicrobial environment. *ISME J* 2022; **16**: 1694–1704.

12. Carver T, Harris SR, Berriman M, Parkhill J, McQuillan JA. Artemis: An integrated platform for visualization and analysis of high-throughput sequence-based experimental data. *Bioinformatics* 2012; **28**: 464–469.

13. Ruis C, Weimann A, Tonkin-Hill G, Pandurangan AP, Matuszewska M, Murray GGR, et al. Mutational spectra analysis reveals bacterial niche and transmission routes. *bioRxiv* 2022.

14. Altschul SF, Gish W, Miller W, Myers EW, Lipman DJ. Basic local alignment search tool. *J Mol Biol* 1990; **215**: 403–410.

15. Freschi L, Jeukens J, Kukavica-Ibrulj I, Boyle B, Dupont MJ, Laroche J, et al. Clinical utilization of genomics data produced by the international *Pseudomonas aeruginosa* consortium. *Front Microbiol* 2015; **6**: 1036.

16. Hmelo LR, Borlee BR, Almblad H, Love ME, Randall TE, Tseng BS, et al. Precision-engineering the *Pseudomonas aeruginosa* genome with two-step allelic exchange. *Nat Protoc* 2015; **10**: 1820–1841.

17. Huang W, Wilks A. A rapid seamless method for gene knockout in *Pseudomonas aeruginosa*. *BMC Microbiol* 2017; **17**.

18. Benjamini Y, Hochberg Y. Controlling the false discovery rate: A practical and powerful approach to multiple testing. *J R Stat Soc Ser B* 1995; **57**: 289–300.

19. Storey JD. The positive false discovery rate: A Bayesian interpretation and the q-value. *Ann Stat* 2003; **31**: 2013–2035.
